# Surface-Doped Zinc Gallate Colloidal Nanoparticles Exhibit pH-Dependent Radioluminescence with Enhancement in Acidic Media

**DOI:** 10.1021/acs.nanolett.3c01363

**Published:** 2023-07-03

**Authors:** Navadeep Shrivastava, Jessa Guffie, Tamela L. Moore, Burak Guzelturk, Amar S. Kumbhar, Jianguo Wen, Zhiping Luo

**Affiliations:** † Department of Chemistry, Physics and Materials Science, Fayetteville State University, Fayetteville, North Carolina 28301, United States; ‡ X-ray Science Division, Argonne National Laboratory, Lemont, Illinois 60439, United States; § Chapel Hill Analytical and Nanofabrication Laboratory, University of North Carolina, Chapel Hill, North Carolina 27599, United States; ∥ Center for Nanoscale Materials, Argonne National Laboratory, Lemont, Illinois 60439, United States

**Keywords:** bioimaging, colloidal nanoparticle, pH, radioluminescence, zinc gallate

## Abstract

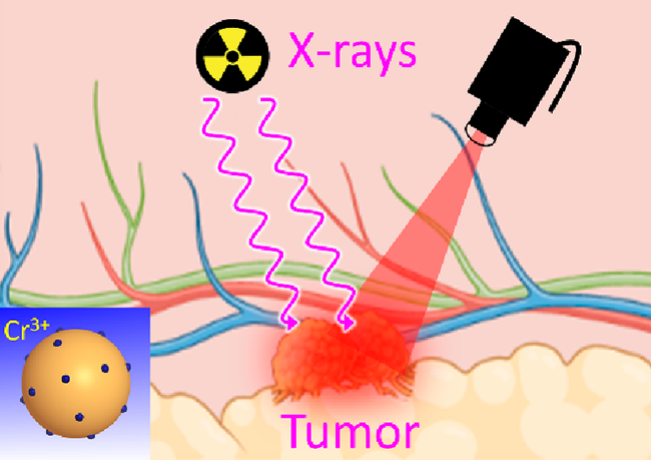

As abnormal acidic pH symbolizes dysfunctions of cells, it is highly desirable to develop pH-sensitive luminescent materials for diagnosing disease and imaging-guided therapy using high-energy radiation. Herein, we explored near-infrared-emitting Cr-doped zinc gallate ZnGa_2_O_4_ nanoparticles (NPs) in colloidal solutions with different pH levels under X-ray excitation. Ultrasmall NPs were synthesized via a facile hydrothermal method by controlling the addition of ammonium hydroxide precursor and reaction time, and structural characterization revealed Cr dopants on the surface of NPs. The synthesized NPs exhibited different photoluminescence and radioluminescence mechanisms, confirming the surface distribution of activators. It was observed that the colloidal NPs emit pH-dependent radioluminescence in a linear relationship, and the enhancement reached 4.6-fold when pH = 4 compared with the colloidal NPs in the neutral solution. This observation provides a strategy for developing new biomaterials by engineering activators on the nanoparticle surfaces for potential pH-sensitive imaging and imaging-guided therapy using high-energy radiation.

pH value plays a pivotal role in modulating cellular behaviors, including cell metabolism, proliferation, apoptosis, vesicle trafficking, etc. An abnormal acidic pH symbolizes the dysfunctions of cells and diseases, such as cancer, Alzheimer’s, and other neurodegenerative diseases.^1−4^ Acid pH also increases the severe acute respiratory syndrome coronavirus 2 (SARS-CoV-2) infection.^5,6^ Sensitive acidity detection and accurate pH measurements are highly desirable in molecular and biomedical research.

Fluorescence-based approaches for pH detection have been developed, which depend on fluorescence signals using nanoprobes.^7−10^ However, their in vivo applications are limited due to the low penetration depths compared with clinically approved high-energy excitations such as X- or γ-rays for imaging or therapeutics.

Recently, nanophosphors have been explored as agents in radiological applications, especially in vivo tumor therapeutics.^11−13^ The combination of insignificant scattering of X-rays in tissues and the high tissue penetration of near-infrared (NIR) optical photons emitted from the phosphors open a pathway of achieving deep tissue optical imaging in vivo with unprecedented high spatial resolution.^14^ Chen et al. designed pH-responsive luminescent Gd_2_O_3_S:Tb core/shell nanocapsules that could release doxorubicin drug in an acidic media, which is applicable for cancer therapy due to the low-pH environment in tumors.^15^ With an almost free background, X-ray radioluminescence (RL) showed enhanced intensity in the low-pH medium, possibly because of optical absorption of the luminescent material or energy transfer. Using NaGdF_4_:Eu^3+^ NPs, Sudheendra et al. found that the X-ray RL decreased with a decrease in pH of the NP solution, possibly due to the surface-coated molecules.^16^ It has been reported that pH sensor films could be used at specific locations to indicate the pH level using X-rays,^17,18^ while a pH-responsive solution is of practical significance for in vivo application, ideally with RL enhancement at a low pH value using X-rays, since the enhanced RL would be an indicative measure of pH and a guide to locate the target for therapeutic imaging. In this work, we report the first observation of pH-dependent enhanced X-ray RL using Cr-doped zinc gallate ZnGa_2_O_4_ (ZGO) colloidal NPs in low-pH acidic media.

The zinc gallate, a cubic spinel oxide, is an appealing host material for a broad range of optical and biological applications.^19^ Cr-doped ZGO (ZGO:Cr) NPs are persistent luminescent nanoparticles (PLNPs) with long-lasting emissions, emitting NIR around 700 nm with high brightness that is suitable for in vivo imaging, as it corresponds to a transmission maximum for biological tissues.^20,21^ The ZGO-based materials produced by traditional solid-state reactions or sol–gel methods require subsequent calcination, causing large particles that are unsuitable for biological applications. In recent years, solution synthesis appears promising, since the samples are only heated in the moderate temperature range of 150–300 °C, with controllable sub 10 nm size with narrowed size distribution.^22−25^ High-energy excitation is used to study the RL of these materials, although limited compared to PL. Song et al. synthesized Cr- and W-codoped ZGO via a hydrothermal method and observed higher luminescence intensity upon X-ray excitation since W improved the X-ray photon absorption efficiency and provided additional electrons to Cr^3+^.^26^ On the other hand, Beke et al. synthesized a ZGO/SiC core/shell structure with a size of 9–9.5 nm and observed that the inclusion of SiC in the core could enhance X-ray RL, as SiC was also excited by X-rays that provided electrons transferred to Cr^3+^ to enhance the luminescence.^27^ Despite the high performances of the solution-synthesized ultrasmall NPs, the detailed structure of the NPs regarding the dopant distribution is unknown, and research on their luminescence properties related to particle size and processing requires much exploration.

In this work, we present a well-controlled synthesis of ZGO:Cr NPs with activator Cr^3+^ ions located on the nanoparticle surface (Figure 1a), and we demonstrated that such ultrasmall NPs have the potential for biomedical applications (Figure 1b). When X-rays interact with NPs, different X-rays (transmitted, Compton scattered, coherent scattered, and fluorescent), electrons (secondary and Auger), holes, and other signals are produced (Figure 1c). We found that the luminescence mechanism by this interaction confirms the activator distribution on surface of NPs. Further, our prepared colloidal NP solutions form smaller aggregates in a neutral solution while larger aggregates form in acidic solutions (Figure 1d,e). Experimentally, the RL from the colloidal solutions exhibits a novel pH dependence with a linear relationship, and the acidic medium enhances the luminescence intensity by 4.6-fold at pH = 4 in comparison to a neutral solution with pH = 7.2. Since a low pH value of the acidic medium is an indication of cell dysfunction or disease, such as tumors, this observation indicates potential applications of the PLNPs with activators on the surface for bioimaging or imaging-guided therapy upon high-energy activation.

**Figure 1:**
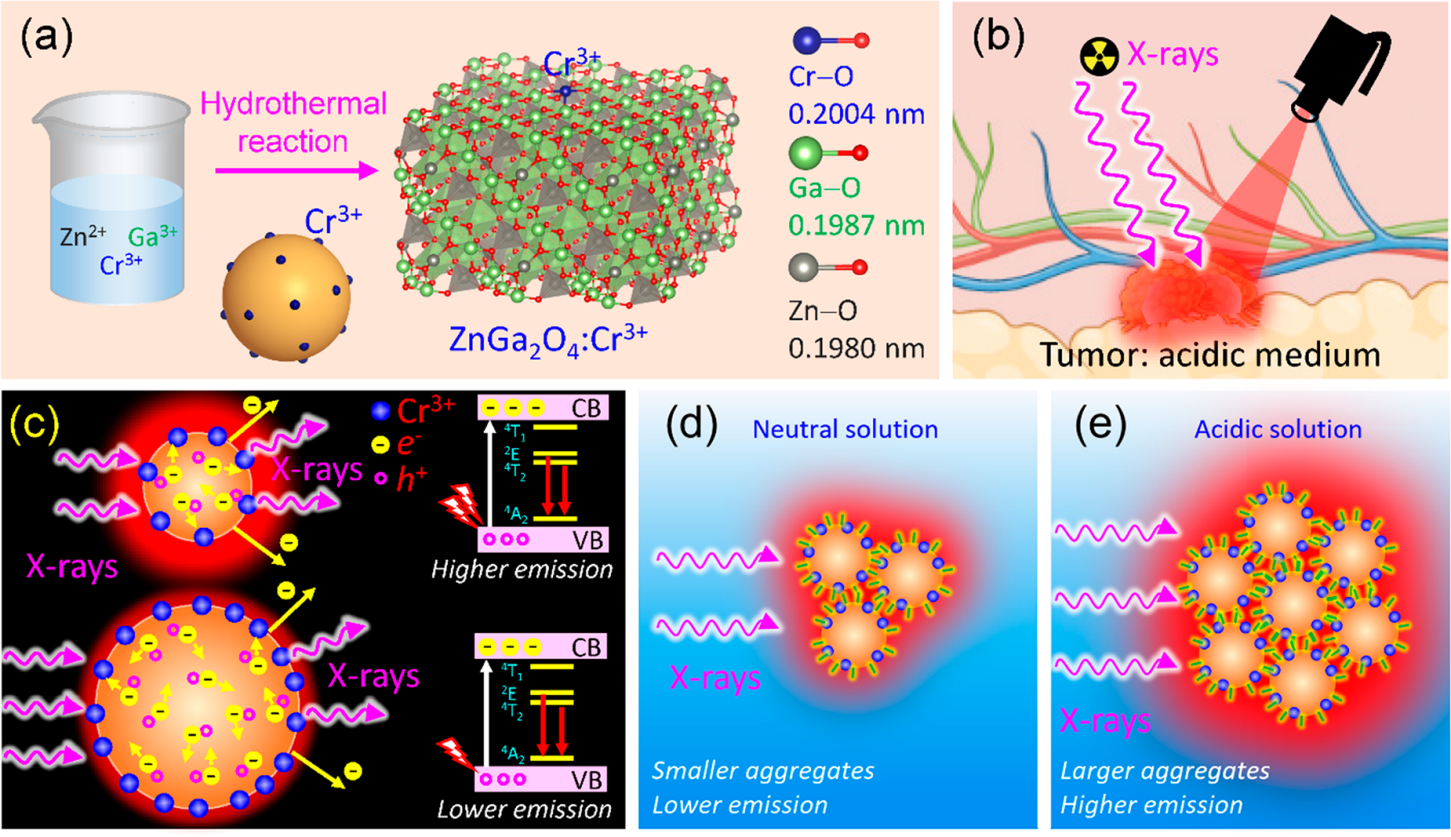
(a) Synthesis of ZGO:Cr NPs. The activator Cr^3+^ is located on the surface of NPs, and the bond lengths are taken from ref (28). (b) Detection of tumor cells in an acidic environment with low pH. (c) X-ray interaction with smaller and larger NPs. (d, e) Smaller colloidal aggregates are formed in neutral solution, while larger aggregates form in acidic solutions, producing enhanced emission upon X-ray excitation.

The hydrothermal method yields high-crystallinity and highly luminous NPs with easy control of the NP size. With the low addition of 1.75 vol % of NH_4_OH precursor in the synthesis, heating at 180 °C for 4, 8, and 20 h yields NPs with diameters of 6.1, 8.1, and 11.5 nm, respectively, while with 2.55% and 3.29% of NH_4_OH in the synthesis, heating at the same temperature for 20 h yields NPs with increased size of 14.2 and 18.5 nm, respectively (Figures S1 and S2). The higher concentration of NH_4_OH promoted the growth of the ZGO lattice according to eqs S2–S4.

The ZnGa_2–*x*_Cr_*x*_O_4_ sample synthesized with a low NH_4_OH concentration of 1.75% for a short time of 4 h has the smallest diameter, which exhibits the highest performance in the RL when *x* = 0.01 (denoted as ZGO:0.01Cr thereafter). We first present a characterization of this sample using transmission electron microscopy (TEM) and first-principles density functional theory (DFT) calculations. An X-ray diffraction (XRD) pattern of the as-prepared NPs is shown in Figure 2a, which matches the standard 04-19-5774. The Rietveld refinement reveals a pure phase with a lattice parameter of *a* = 0.8386(3) nm. Figure 2b shows a selected-area electron diffraction (SAED) with poly rings. Intensity profiles of the reflections are produced from the center beam, and after subtracting the high background, the profiles are similar to the XRD pattern.^29^Figure 2c shows the compositional analysis by X-ray energy-dispersive spectroscopy (EDS). An evident Cr peak is identified, and the quantitative analysis is consistent with the normal composition within experimental errors (the Fe peak is from the polepiece of the instrument). Figure 2d displays a TEM image at lower magnification, showing the uniform distribution of the synthesized NPs with an average size of 6.1 nm. In the high-resolution TEM (HRTEM) image in Figure 2e, NPs are shown in a near-spherical shape, and lattice fringes with a spacing of 0.3 nm are observed, corresponding to the (220) plane of ZGO. A high-angle annual dark-field (HAADF) image in scanning TEM (STEM) mode is taken, as shown in Figure 2g. Elemental mapping by EDS in the STEM mode is conducted using a defined electron beam spot size of less than 1 nm. The elements of Ga (Figure 2h), Zn, and O (Figure S3) exhibit uniform distribution over the NPs, while Cr shows a discontinuous distribution (Figure 2i). After superimposing the HAADF image and Cr map, NP boundaries could be recognized, as shown in Figure 2j, implying that Cr ions are on the surface of NPs.

**Figure 2:**
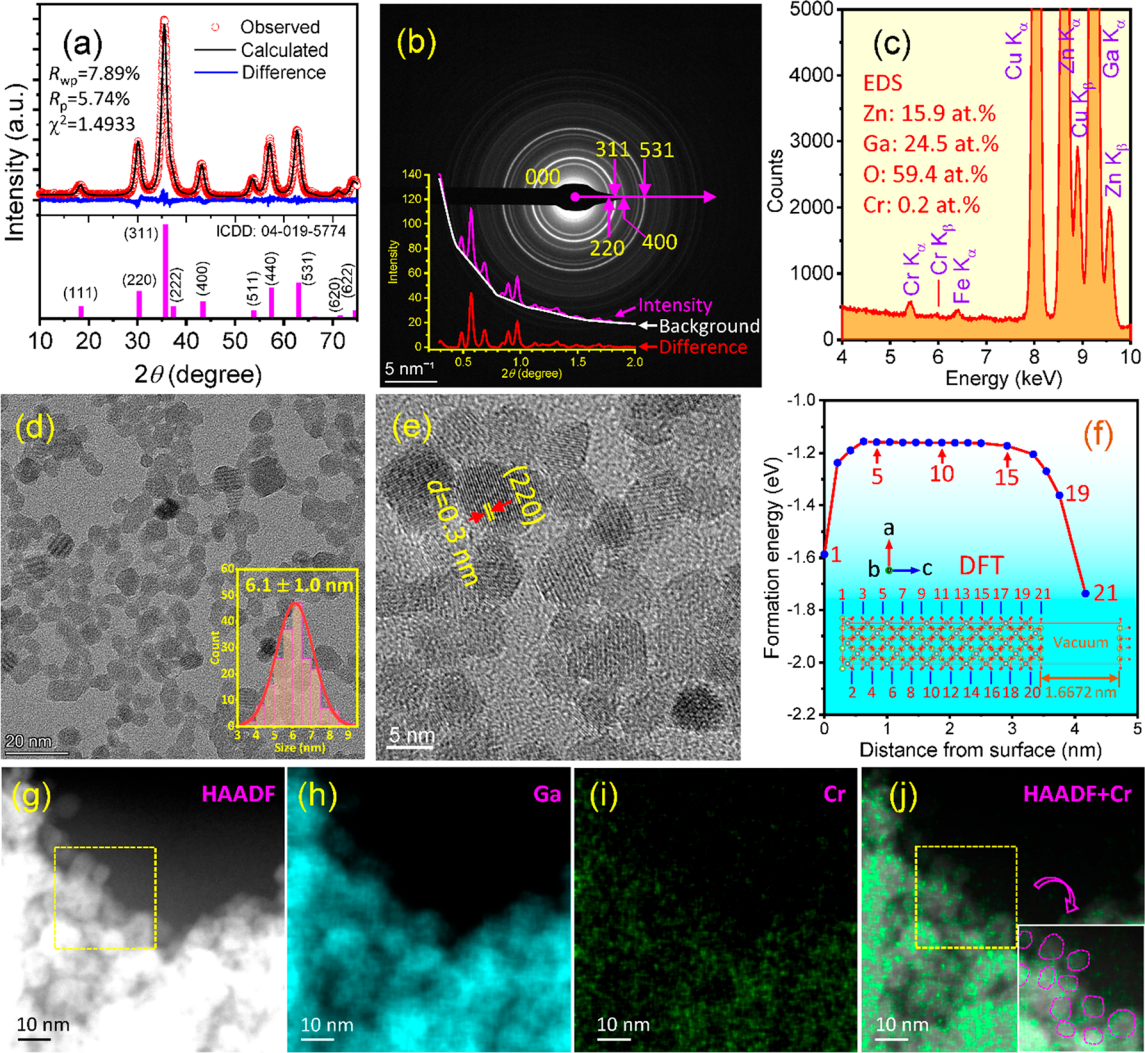
(a) XRD analysis of ZGO:0.01Cr NPs synthesized with 1.75% NH_4_OH and 4 h heating time and corresponding Rietveld refinement. (b) SAED. (c) EDS. (d) TEM. (e) HRTEM. (f) DFT calculations of formation energy of a single Cr on different layers from the surface to volume, showing the lowest energy when it resides on the surface. (g) HAADF image. (h, i) EDS maps of Ga and Cr, respectively. (j) superimposed HAADF and Cr map, showing the distribution of Cr on the surface of NPs. Corresponding areas are framed in (g) and (j).

DFT calculations are conducted to confirm the formation of the Cr dopant on the surface. A recent work indicated that the Cr–O bond length is significantly longer than those of Ga–O and Zn–O, although Cr^3+^ is slightly smaller than Ga^3+^.^28^ Here, we evaluate the stability of Cr on the location of the crystal from the surface to volume by selecting a slab of 1 × 1 × 5 supercells with a vacuum space in 1.6672 nm isolating the crystal lattices (inset in Figure 2f). Ga is on the termination of the crystal surface. The formation energy *E*_f_ of the Cr dopant is given by
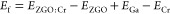
1where *E*_ZGO:Cr_, *E*_ZGO_, *E*_Ga_, and *E*_Cr_ represent the total energies of Cr-doped ZGO, undoped ZGO, pure element Ga, and pure element Cr, respectively. The total energies are calculated by using the *Quantum ESPRESSO* program, and the detailed results are given in Table S1. We only replace one Ga with Cr at the 21 locations, as indicated in the inset in Figure 2f. The computed *E*_f_ is plotted in Figure 2f. It is found that, on the crystal surface, *E*_f_ is significantly lower than the locations in the volume, indicating that Cr is more stable on the surface compared with its location in the volume. With a low concentration of NH_4_OH which leads to a slow growth rate of ZGO, during the slow furnace cooling process, Cr^3+^ ions attend the reactions last at lower temperatures so that they appear on the surface, while with a higher concentration of NH_4_OH which leads to a fast growth rate of ZGO, or by a fast reaction route like a coprecipitation method, as the reactions occur quickly, Cr^3+^ ions can grow inside the NPs.

The PL emission spectra of ZnGa_2–*x*_Cr_*x*_O_4_ synthesized under conditions of 1.75% NH_4_OH and 4 h heating time are shown in Figure 3a with different Cr^3+^ doping levels (*x* = 0.005, 0.01, 0.02, and 0.05), while the excitation spectra are shown in Figure S4a. The undoped ZGO sample showed a broad band at 350–620 nm with a maximum peak around 455 nm (^2^E_B_ → ^4^A_2_), which is caused by self-activation originating from the partial substitution of the Zn^2+^ site by Ga^3+^ in the spinel structure.^30^ The predominant Cr^3+^ emission comes from the ^2^E → ^4^A_2_ transition. The optimum concentration is found to be 0.01Cr, and beyond this composition, the emission intensity is reduced by concentration quenching.

**Figure 3:**
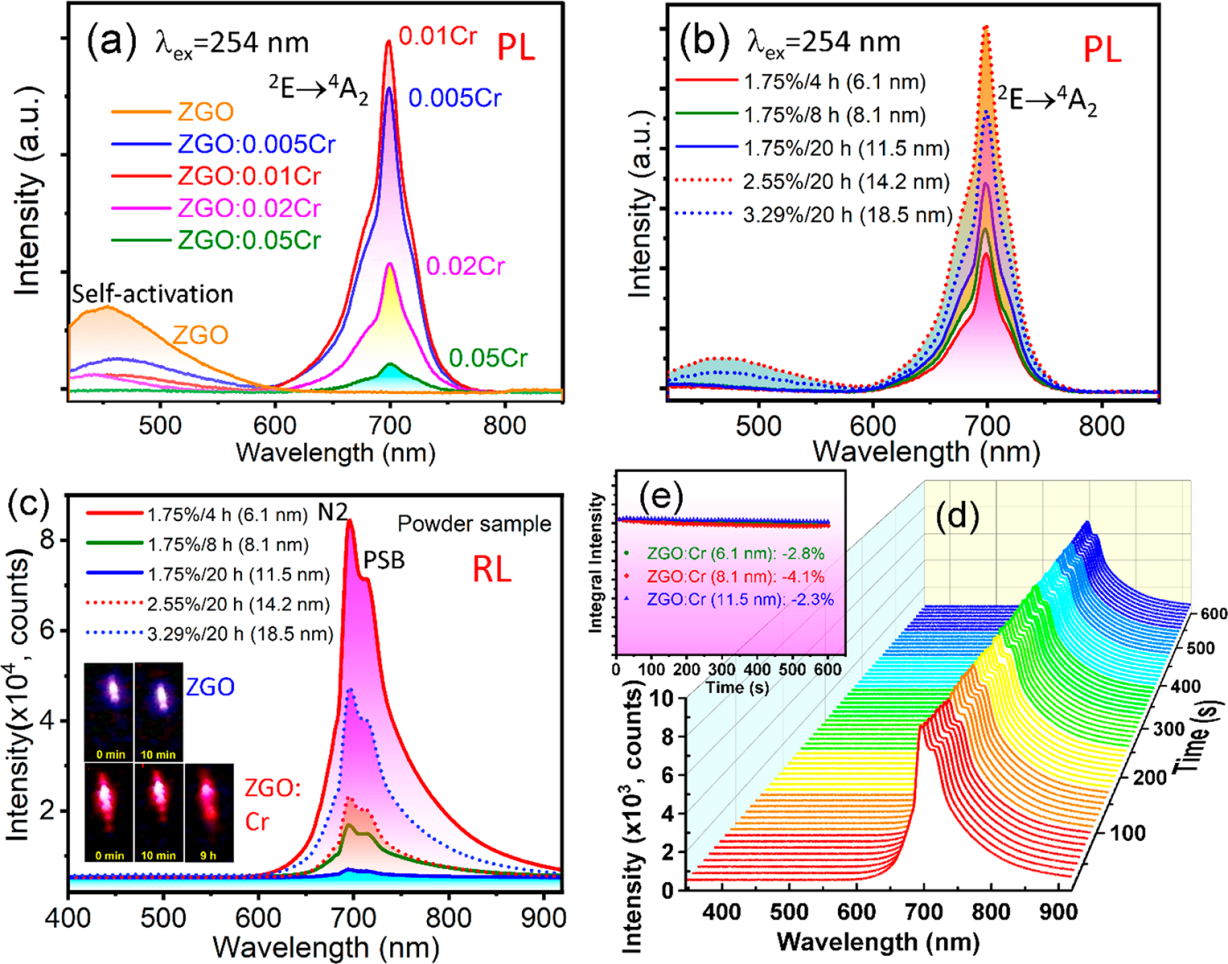
(a) PL emission spectra with different Cr^3+^ doping levels of ZnGa_2–*x*_Cr_*x*_O_4_ (*x* = 0.005, 0.01, 0.02, and 0.05) synthesized under 1.75% NH_4_OH and 4 h heating time; (b, c) PL and RL emission spectra of ZGO:0.01Cr NPs synthesized under varied NH_4_OH concentrations and heating time as indicated; (d) time-resolved RL stability test of ZGO:0.01Cr NPs (6.1 nm by 1.75%/4 h) under the maximum energy flux of X-rays for 600 s; and (e) integrated intensity showing high stabilities.

The PL spectra of ZGO:0.01Cr, synthesized under varied NH_4_OH concentrations and heating times, are presented in Figure 3b, showing the size effect. Their excitation spectra are shown in Figure S4b. It is found that as the particle size increases, the PL emission also increases, possibly because of reduced surface areas where surfactants of hydroxyl radicals are attached, acting as quenchers.^31^ From the Fourier transform infrared (FTIR) spectrum in Figure S5, the presence of hydroxyls is evident. It was recently reported that subsequent annealing of as-synthesized 10 nm NPs at 800 °C increased the PL emission intensity, possibly related to the removal of surfactants at the high temperature and redistribution of Cr ions for optimum emission.^25^ However, after high-temperature annealing, the NPs are severely aggregated.

The RL spectra of these samples are shown in Figure 3c. These emission peaks still show the characteristic emissions of Cr^3+^, while the shape of the emission appears as the N2 and phonon sideband (PSB). Note that the emission from the host disappears, indicating a thorough energy transfer from the host to the Cr^3+^ ions. The RL and PL have different luminescence mechanisims.^32^ As shown in Figure 1c, within the NPs, electrons and holes are generated by incoming X-rays. The electrons/holes can reach the surface to excite the activators, while they cannot travel a long distance in the lattice due to their limited energy (estimated to be less than 1 nm for energy up to 200 eV). Thus, as the particle size increases, a larger portion of electrons and holes are annihilated in the volume, and they do not contribute to the excitation; thus, these NPs, with the same 1.75% NH_4_OH addition, show reduced emissions as the size increases. However, with higher concentrations of NH_4_OH, as the NH_4_OH promoted the crystal growth, the activators incorporated in the particle volume instead of on the surface; more activators could be excited by X-ray excited electrons and holes in the volume to enhance the RL. Note that the X-rays and electrons that escaped from the particles can also excite the particles nearby by multiple scattering, causing even higher emission if the activators are on the surface. Therefore, both the PL and RL data support the activator surface distribution on the NPs synthesized with a low NH_4_OH concentration.

The emission stability of ZGO:0.01Cr NPs (6.1 nm in diameter) irradiated with X-rays at a maximum fluence for 10 min is shown in Figure 3d. No emissions appear in the range of 360–600 nm, while emissions from the ^2^E → ^4^A_2_ transition of Cr^3+^ (∼696 nm) are almost steady without any peak shifts over the tested period. A stability test of emission spectra of the ZGO host is shown in Figure S6. Figure 3e plots the integral intensities (area under the curve), which are almost stable with a small reduction from 2.3–4.1% that is better than the stability of a halide.^33^ Photos of the tested samples are inserted in Figure 3c, where undoped ZGO exhibits a blue color and ZGO:Cr exhibits a red color, over the long-term irradiation.

Colloidal solutions are prepared by dispersing ZGO:0.01Cr (6.1 nm synthesized under 1.75% NH_4_OH and 4 h heating) NPs in phosphate-buffered saline (PBS) solutions with different pH values at an optimum concentration of 3 mg/mL. The UV–vis absorption spectra are shown in Figure S7. The PL emission spectra of colloidal solutions are recorded as shown in Figure 4a, and excitation spectra are shown in Figure S8. Note that the surfactants on the NP surfaces absorb energy and the absorption peak energy in Figure S7 is higher than that of the excitation energy in Figure S8, while the solution with pH = 4 shows the highest absorption in Figure S7. All luminescence measurements are performed using a fresh aliquot of NPs in the PBS at pH = 7.2, 6.5, 6, 5, and 4. The PL shows emission at 696 nm due to the ^2^E → ^4^A_2_ transition, whereas the emission from water is negligible. In addition, self-activation is pronounced. The process involves an energy transfer from the Ga^3+^ ion placed in the octahedral sites toward its first six neighbors.^34^ This blue emission exists in all specimens at different pH values but is lower than that of the NPs in the water-like medium. As shown in the decay curves in Figure S9, the emission lifetime is in the millisecond range. With an increase in acidity, the average lifetime increases, indicating more surface defects on larger aggregates. Lowering the pH from 7.2 to 4 with increased acidity yields a slight increase in the red emission. A close observation indicates a slight enhancement of the emission as the acidity increases (inset in Figure 4a). This observation suggests that the UV energy is not sufficient to excite the colloidal NPs, as the emission from the host is significant and is not thoroughly transferred to the Cr^3+^ ions as it is in the powders, and thus energetic X-rays may be used to examine the colloidal solutions.

**Figure 4:**
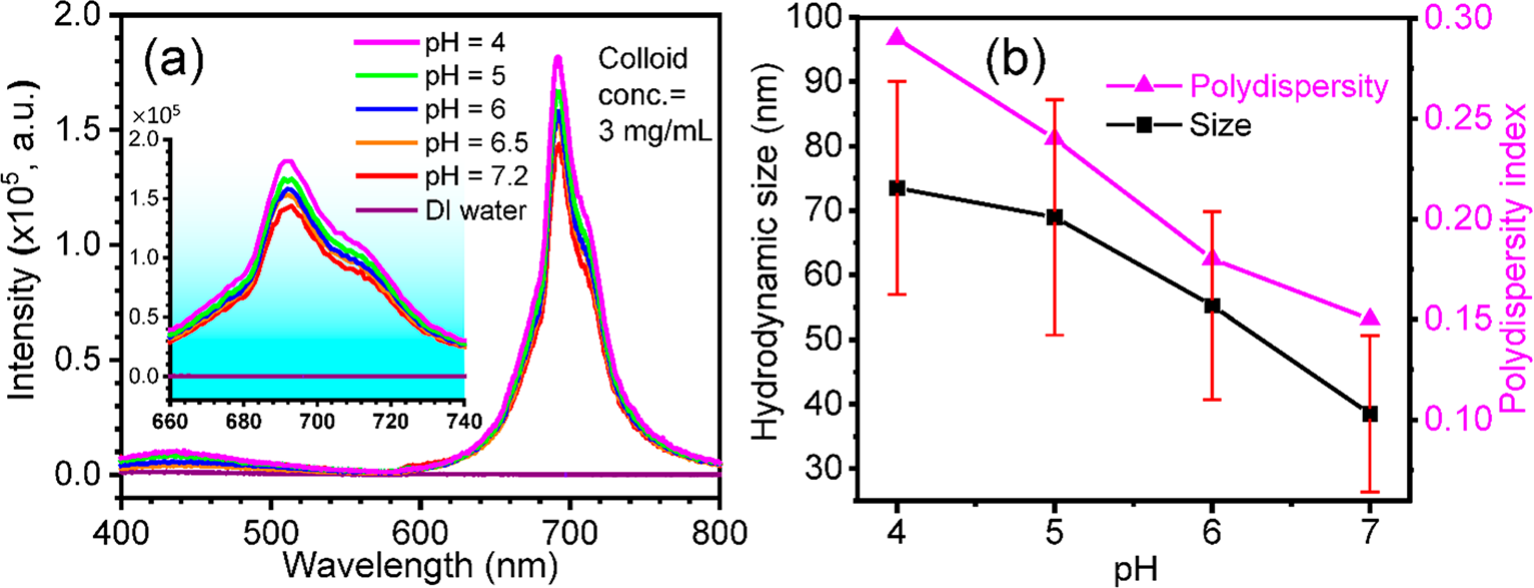
(a) PL emission spectra of ZGO:0.01Cr (6.1 nm) colloidal NPs in different pH buffer solutions (pH = 7.2, 6.5, 6, 5, 4) and (b) hydrodynamic size and PDI versus pH of the colloidal solution.

Dynamic light scattering (DLS) and zeta (ζ) potential experiments are performed to study the colloidal solutions. As shown in Figure 4b and Table S2, hydrodynamic sizes are found to be in the range from 38.2 to 73.5 nm. At neutral pH = 7.2, the measured distribution data have a very intense peak around 38.2 nm. The polydispersity index (PDI) is around 0.15, suggesting monodispersed colloidal particles. As shown in Figure 4b, the hydrodynamic diameter increases in acidic media when the pH is reduced from 7.2 to 4, indicating the formation of larger aggregates causing multiple scattering effects to enhance the emission. In the acidic medium, the surface negative charges of nanoparticles with hydroxyl radicals are reduced, affecting the aggregate equilibrium formed in the neutral solution because of reduced repulses, resulting in a larger aggregation. The PDI also increases with the reduction of pH. The ζ potential measurements reveal the role of surface charge and interactions at different PBS-buffer solutions.^35^ The ζ potentials are given in Table S2. The ζ potential indicates the dispersion stability of particles in colloids, ranging from 36.1 to 52.2 mV when the pH is reduced from 7.2 to 4. The pH has a pronounced effect on the ζ potentials. The intense charge on the surface increases the charge–charge repulsions between the particles, thus maintaining a stable and monodisperse suspension.

The colloidal solutions are further tested under X-ray excitation. The background emissions are shown in Figure S10, which are negligible to the intensities from the dispersed NPs. The RL spectra are shown in Figure 5a, which display an intense peak at around 693 nm with a few broad shoulders on the side of the near-infrared region. The emission from the host was significantly reduced compared with the PL in Figure 4a, although it is still present. The integral intensities are plotted vs the pH values, as shown in the inset of Figure 5a. The emission increases by 4.6-folds with the acidity when the pH decreases from 7.2 to 4. As illustrated in Figure 1d,e, larger aggregates will show higher emission than smaller aggregates due to the multiple scattering effects and possibly favorable energy transfers between the linked NPs.

**Figure 5:**
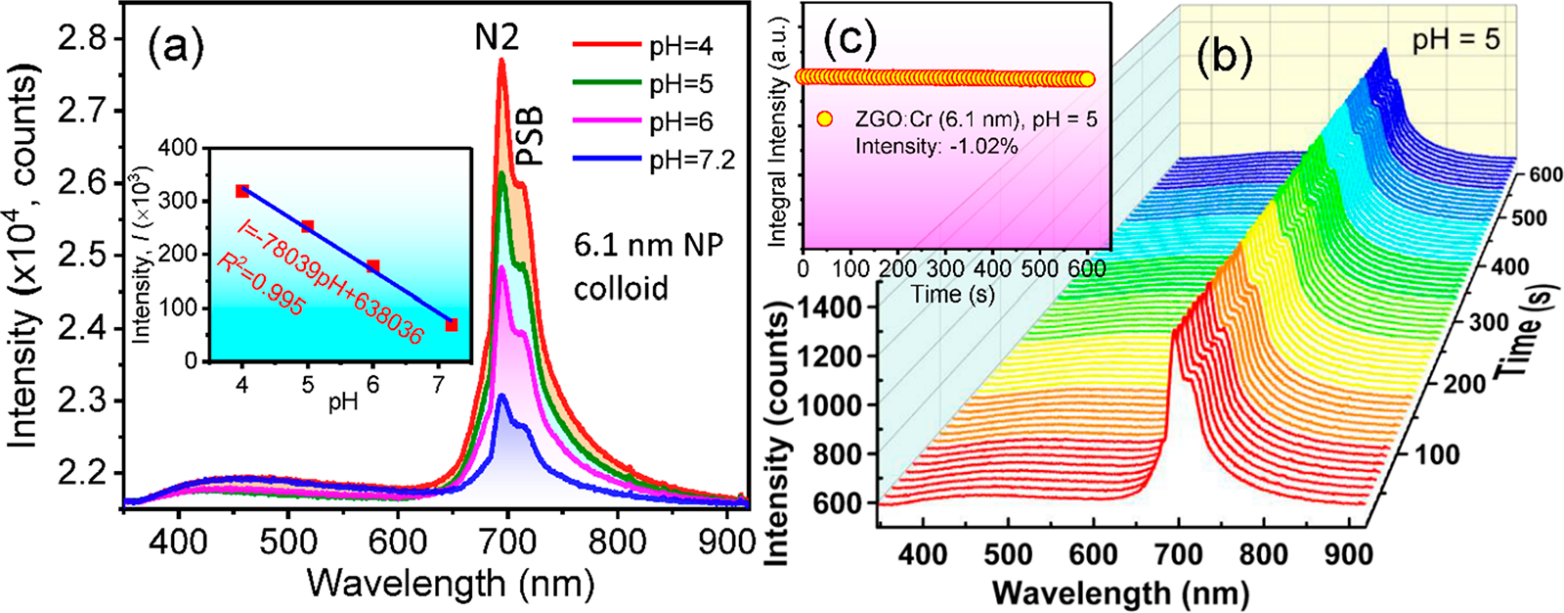
(a) RL emission of colloidal ZGO:0.01Cr (6.1 nm) NPs in different pH solutions, (b) stability test under maximum energy flux of the X-rays, and (e) integrated intensity.

The stability of the colloidal solution with pH 5 is tested, as shown in Figure 5b. Each spectrum was collected every 10 s, and the dispersed NPs in the cuvette were exposed for 10 min. A close observation depicts invariable changes in the emission intensity at 696 nm, clearly suggesting that NPs remained dispersed and emission centers were not settled. The integrated intensity measurements show that the colloidal intensity is reduced by only 1.02% (Figure 5c), which is even less than that of the powder samples (Figure 3e). This experiment demonstrated that the experimental materials have the potential for applications in pH-sensitive cells, where pH is a crucial factor in developing novel therapeutics such as nanoparticle-assisted X-ray photodynamic therapy. The sensitive detection of pH through the X-ray emissions can provide a general guide to distinguish local disease intracellular environment through the pH-responsive agents.

## Data Availability

The data that support the plots in the manuscript are available from the corresponding authors upon reasonable request.

## Supporting Information Available

Experimental details, XRD, TEM, EDS, PL, FTIR, X-ray irradiation stability, UV–vis absorption, excitation, decay curves, RL, DFT, ζ potential, DLS, and DLS-PDI (PDF)
