# Supplementary material for: Surface-Doped Zinc Gallate Colloidal Nanoparticles Exhibit pH-Dependent Radioluminescence with Enhancement in Acidic Media
Source: Nano Lett. 2023 Jul 3;23(14):6482–8. doi: 10.1021/acs.nanolett.3c01363 (PMC10375584; doi:10.1021/acs.nanolett.3c01363)
Supplement: Supplementary file 1 — nl3c01363_si_001.pdf [file nl3c01363_si_001.pdf]

Supplementary Information

Surface-Doped Zinc Gallate Colloidal Nanoparticles Exhibit pH-Dependent Radioluminescence with Enhancement in Acidic Media

Navadeep Shrivastava [a], Jessa Guffie [a], Tamela L. Moore [a], Burak Guzelturk [b], Amar S. Kumbhar [c], Jianguo Wen [d], and Zhiping Luo [a*]

[a] Department of Chemistry, Physics and Materials Science, Fayetteville State University, Fayetteville, North Carolina 28301, United States

[b] X-ray Science Division, Argonne National Laboratory, Lemont, Illinois 60439, United States

[c] Chapel Hill Analytical and Nanofabrication Laboratory, University of North Carolina, Chapel Hill, North Carolina 27599, United States

[d] Center for Nanoscale Materials, Argonne National Laboratory, Lemont, Illinois, 60439, United States

*Email: zluo@uncfsu.edu

EXPERIMENTAL METHODS

Materials. All prepared samples were synthesized from $Zn(NO_3)_2$, $Ga(NO_3)_3{\cdot}xH_2O$, $Cr(NO_3)_3{\cdot}9H_2O$, NH_4OH, and de-ionized (DI) water. Absolute ethanol and HCl-water were used during the washing process.

Synthesis. For the $ZnGa_2O_4$ host, 10 mL $Ga(NO_3)_2{\cdot}xH_2O$ (0.2 M) and 5 mL $ZnCl_2$ (0.2 M) were mixed in a 50 mL flask by stirring. Ammonium hydroxide NH_4OH (28%) solution in 1 mL was quickly added into the flask (the NH_4OH concentration is 1.75 % in volume). After 30 min of stirring, the mixture was transferred to a Teflon-lined autoclave (50 mL in volume). After reaction at 180 °C for 4 h, 8 h, and 20 h, respectively, the system was cooled to room temperature slowly in the furnace. For comparison, to promote the reaction, we also synthesized samples by adding NH_4OH in 1.5 mL and 2 mL to reach 2.55% and 3.29% in volume, respectively, and heated for 20 h. In a sequence, the products were centrifuged and washed two times with ethanol and one time with DI water mixed with 2% HCl. The powder samples were dried at 60 °C for 12 h. To prepare Cr-doped $ZnGa_{2-x}O_4$:xCr (x = 0.005, 0.01, 0.02, 0.05) NPs, the same process was repeated by replacing the equivalent stoichiometric value of $Ga(NO_3)_3{\cdot}xH_2O$ with $Cr(NO_3)_3{\cdot}9H_2O$ and mixing them first before the addition of Zn^{2+} precursor. To prepare colloidal solutions, 3 mg/mL powders were dispersed in acidic PBS solutions with different pH (7.2, 6, 5, and 4), followed with sonication for 15 min.

Instruments. The crystal structure was characterized by using XRD (Rigaku MiniFlex 600) operating at 30 kV and 10 mA, with a step size of 0.02 and a scanning speed of 0.08 deg min^{-1}. TEM was conducted using Thermo Scientific Talos F200X at 200 kV. The PL was conducted using a Shimadzu RF–5301PC Spectrofluorophotometer and Horiba FluoroMax Plus Spectrofluorometer. The PL decay time was measured using Horiba EasyLife L Phosphorescence Lifetime Spectrometer. The FTIR was conducted using Thermo Scientific Nicolet iS50 FTIR spectrometer. The hydrodynamic diameter and zeta potential in suspension were measured by DLS using a Particle analyzer–Litesizer-500 (Anton Paar). Time-resolved RL was conducted using Beamline 11-ID-D in Advanced Photon Source, Argonne National Laboratory, with an X-ray energy of 11.7 keV. Samples were prepared by depositing fine powders on carbon tape on the surface of a glass slide. A single-photon detector iDUS 420 series – Andor spectrophotometer (Oxford Instruments) coupled with pico-quant software was used for emission intensity collection in the range of 350–920 nm. The specimen's standard emission spectra were obtained upon exposure to X-ray irradiation for 0.1 s and averaged over 10 s for powders and 40 spectra (accumulation). The input and output slit widths were 100 and 1800 um, respectively.

DFT calculation. The DFT calculations were made using *Quantum ESPRESSO* package.[1,2] We used the Perdew-Wang (PW91) gradient-corrected correlation functionals in the generalized gradient approximation (GGA). For a higher accuracy, DFT+*U* method was conducted, with the same *U* perturbation values in the reference.[3] The self-consistent convergence is set at 1.36×10^{-5} eV/atom, plane-wave cut-off energy is 285.7 eV, and cut-off for charge is 3,401.4 eV. The Brillouin K-point grid is 1×1×1 for the supercell with a large *c*. The calculated band gap is 4.44 eV, being consistent with the literature report.[3,4] The total energy E_{Ga} is calculated from tetragonal Ga with *a*=0.2813 and *c*=0.4452 nm, and E_{Cr} from cubic Cr with *a*=0.2970 nm.

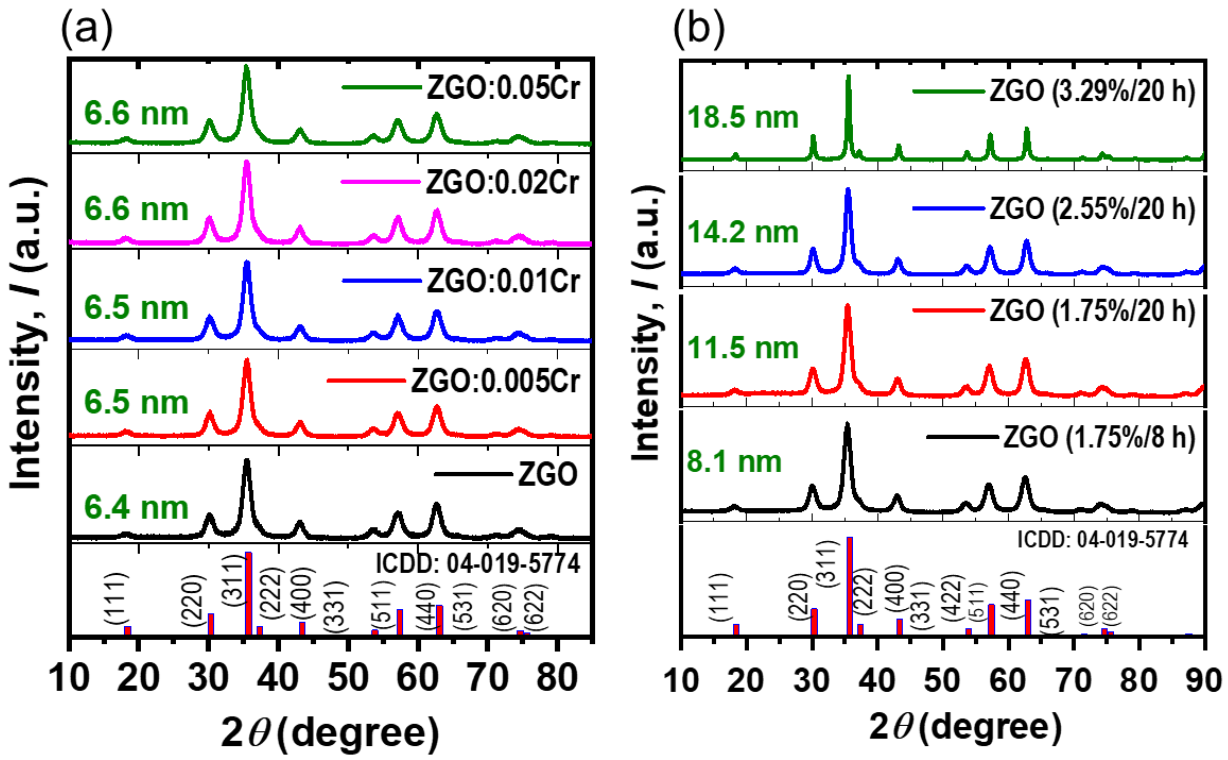

Fig. S1. XRD patterns. (a) $ZnGa_{2-x}Cr_xO_4$ (x=0.005, 0.01, 0.02, and 0.05), synthesized with the addition of 1.75% of NH_4OH and 4 h heating time; (b) $ZnGa_{2-x}Cr_xO_4$: x=0.01, synthesized with varied NH_4OH concentrations and heating time. The NP size determined by the Scherrer equation as follows:

$$t = \frac{0.89\lambda}{B\cos\theta_{\mathrm{B}}}, \qquad \text{(Eq. S1)}$$

where λ is the X-ray wavelength, B is the full width at half max (FWHM) of the Bragg peak, and θ_{B} is the Bragg angle. It is found that a longer heating time yields a larger particle size.

The addition of NH_4OH is needed in the following reactions:

$$\mathrm{Zn^{2+} + 4OH^- = Zn(OH)_4^{2-}}, \qquad \text{(Eq. S2)}$$

$$\mathrm{2Ga^{3+} + 8OH^- = 2Ga(OH)_4^-}, \qquad \text{(Eq. S3)}$$

$$\mathrm{Zn^{2+} + 2Ga^{3+} + 12OH^- = ZnGa_2O_4 + 4H_2O + 4OH^-}. \qquad \text{(Eq. S4)}$$

It is found that adding higher concentration of NH_4OH promotes the reactions, so the crystals grow faster. When the NH_4OH concentration is raised from 1.75% to 2.55% and 3.29% with the same heating time of 20 h, crystal size increases from 11.5 nm to 14.2 nm and 18.5 nm, respectively.

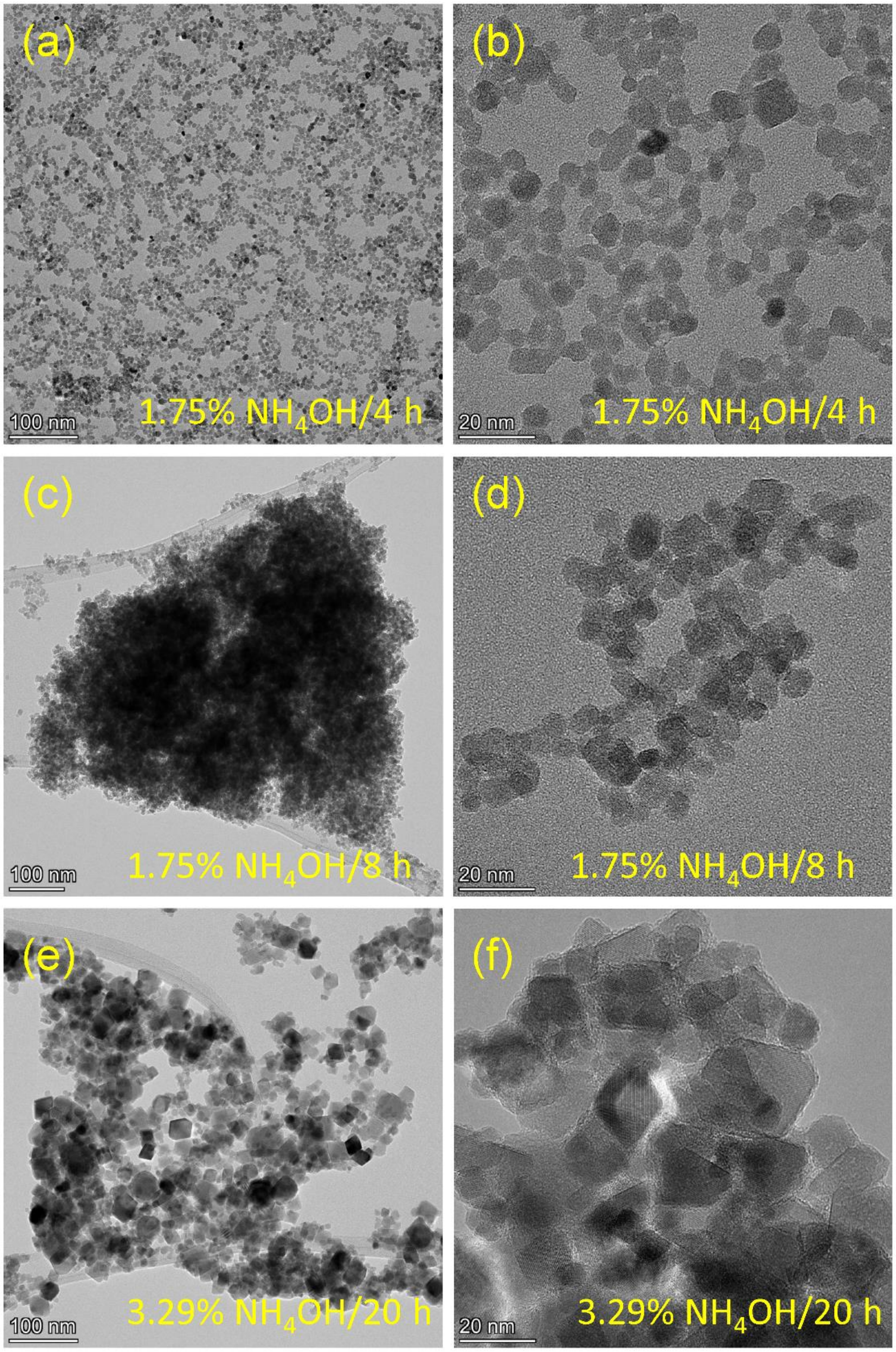

Fig. S2. Representative TEM image of $ZnGa_{2\text{-}x}Cr_xO_4$ (x=0.01) synthesized under conditions with different reaction conditions of (a, b) 1.75% NH_4OH and 4 h heating time; (c, d) 1.75% NH_4OH and 20 h heating time; and (e, f) 3.25% NH_4OH and 20 h heating time.

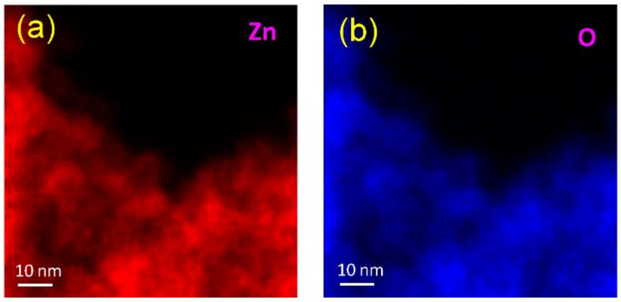

Fig. S3. TEM EDS maps of Ga (a) and O (b) of $ZnGa_{2-x}Cr_xO_4$ (x=0.01) NPs synthesized with 1.75% NH_4OH and dual time of 4 h. The area is the same as Fig. 2g–j.

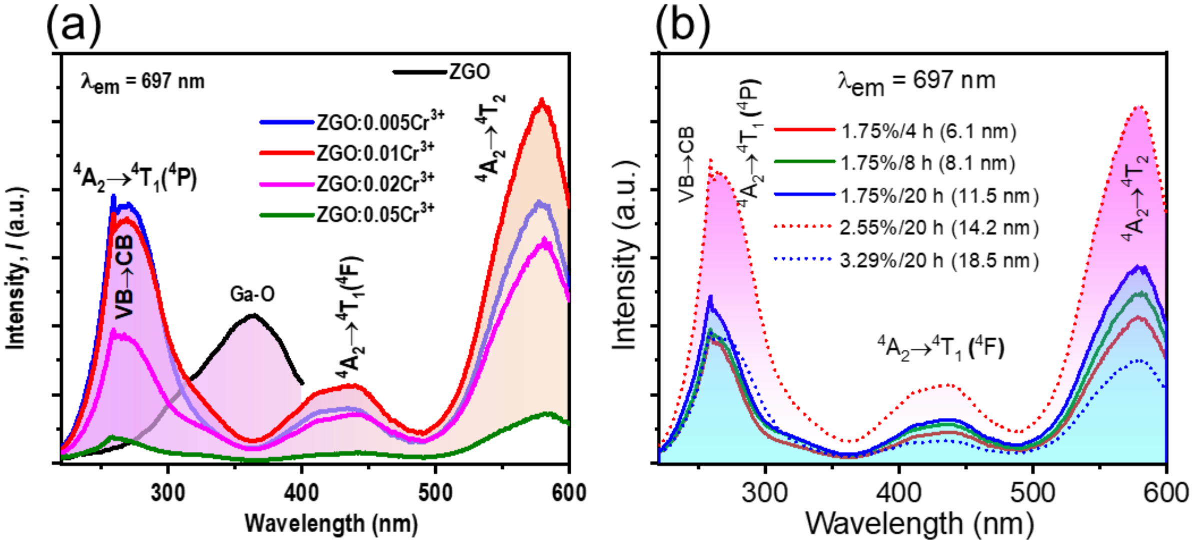

Fig. S4. Excitation spectra for the emission at 697 nm. (a) ZGO host and $ZnGa_{2-x}Cr_xO_4$ (x=0.005, 0.01, 0.02, and 0.05), synthesized with 1.75% NH_4OH and 4 h dual time; (b) $ZnGa_{2-x}Cr_xO_4$: x=0.01, synthesized with varied NH_4OH concentration and dual time.

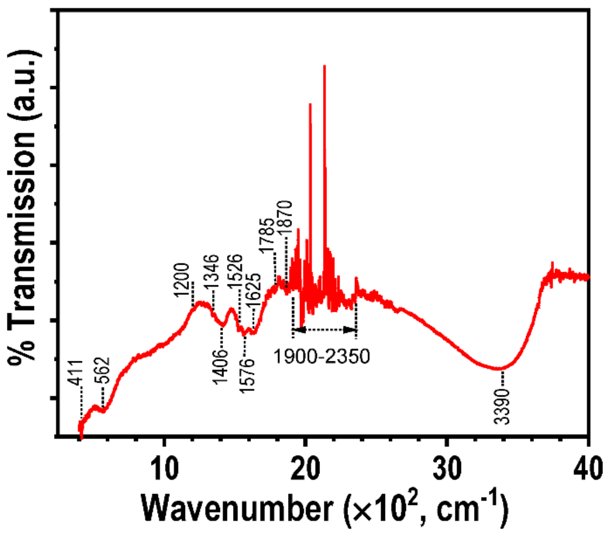

Fig. S5. FTIR spectrum of $ZnGa_{2-x}Cr_xO_4$ (x=0.01) NPs (6.1 nm in diameter) synthesized by hydrothermal reaction. The broad peak at region 3390 cm^{-1} is due to the (O–H) vibration stretching absorbed by the sample, or due to the M–OH–M (where M is a metal). The peak in the range 1400 – 1780 cm^{-1} is due to OH and $-CH_2$ bending of adsorbed moisture in the sample, and 1625 cm^{-1} is due to C=O group. The bands that appeared near 1900 – 2350 cm^{-1} indicate the CO adsorption on the surface of oxide from the environment. The peaks at about 411 cm^{-1} and 562 cm^{-1} represent characteristic metal-oxygen Ga–O and Zn–O vibrations, respectively. The peak at 1406 cm^{-1} corresponds to the adsorbed nitrate ions.

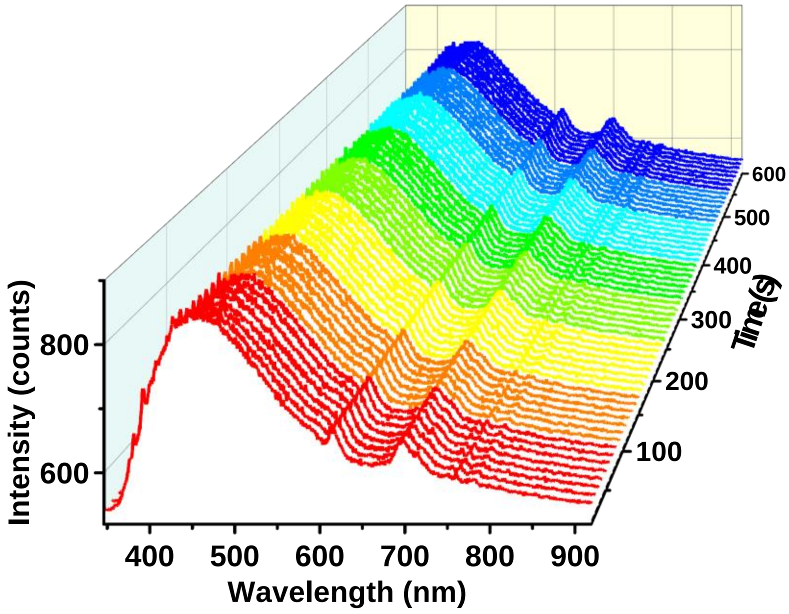

Fig. S6. Stability test against the highest energy fluence of the x-rays prepared of undoped ZGO for 10 min.

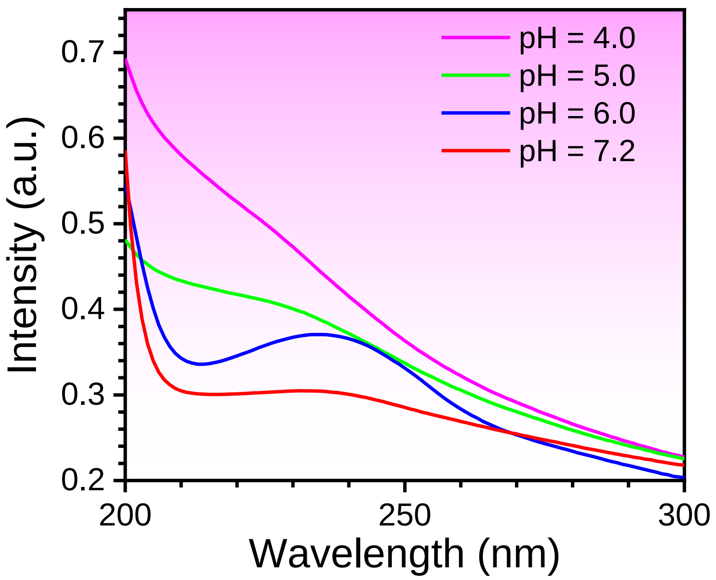

Fig. S7. UV-Vis absorption spectra of ZGO:0.01Cr (6.1 nm) colloidal NPs in different pH buffer solutions.

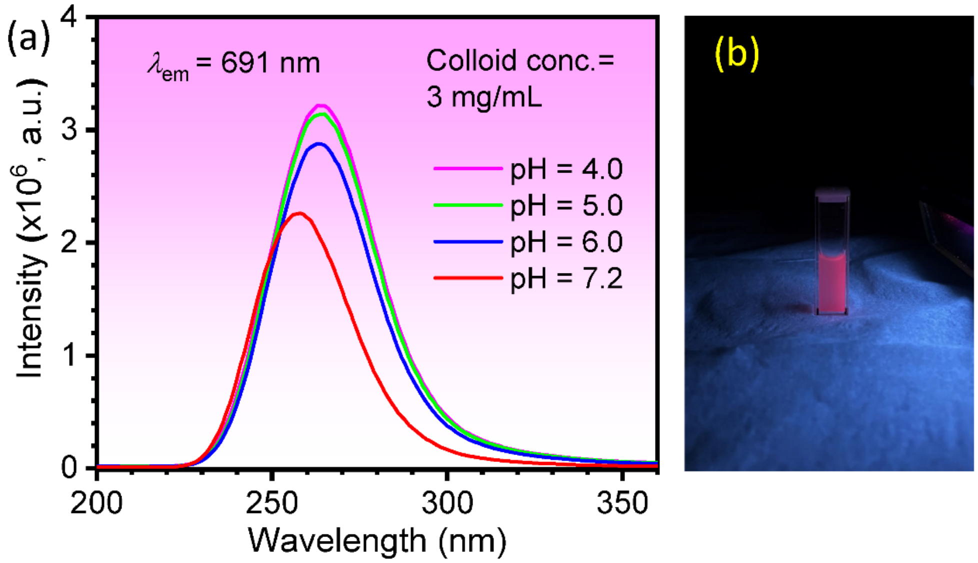

Fig. S8. (a) Excitation spectra of colloidal NPs in different pH buffer solutions, monitored at the 691 nm emission; and (b) a photo of the solution under 254 nm UV excitation.

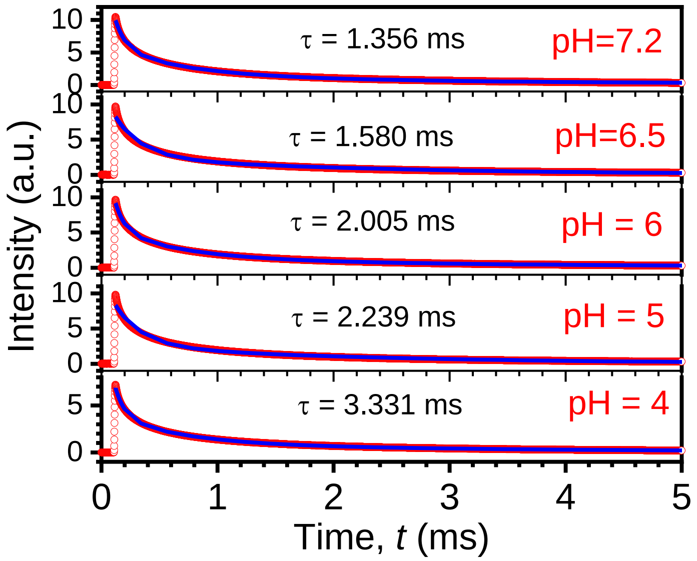

Fig. S9. Decay curves of colloidal NPs in different pH buffer solutions. The decay curves obtained were fitted according to a biexponential decay:

$$I(t) = A_0 + A_1 \exp\left(-\frac{t}{\tau_1}\right) + A_2 \exp\left(-\frac{t}{\tau_2}\right) \quad \text{(Eq. S5)}$$

where $I(t)$ is the intensity at the time t after excitation, A_0, A_1 and A_2 are constants, and τ_1 and τ_2 are fast and slow decay time. The average lifetime is calculated by

$$\tau = \frac{A_1\tau_1^2+A_2\tau_2^2}{A_1\tau_1+A_2\tau_2} \quad \text{(Eq. S6)}$$

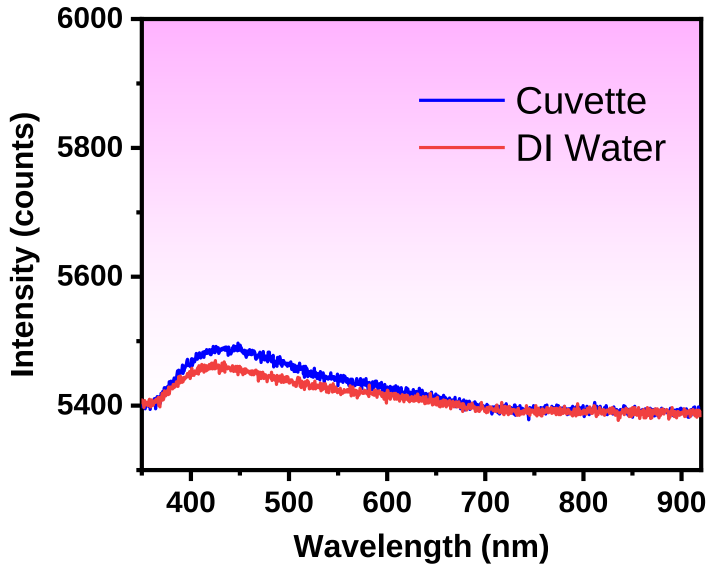

Fig. S10. RL for blank cuvette and cuvette with DI water.

Table S1. DFT calculation results. E_{ZGO}=-356381.25 eV, E_{Ga}=-2423.37 eV, E_{Cr}=-2363.45 eV.

No.	Cr coordinator, *z* (nm)	Distance from the bottom surface (nm)	Total *energy* (eV)	E_f (eV)
1	0.000	0.000	-356322.91	-1.59
2	0.036	0.208	-356322.57	-1.24
3	0.071	0.417	-356322.52	-1.19
4	0.107	0.625	-356322.48	-1.16
5	0.143	0.834	-356322.49	-1.16
6	0.179	1.042	-356322.49	-1.16
7	0.214	1.250	-356322.49	-1.16
8	0.250	1.459	-356322.49	-1.16
9	0.286	1.667	-356322.49	-1.16
10	0.321	1.876	-356322.49	-1.16
11	0.357	2.084	-356322.49	-1.16
12	0.393	2.292	-356322.49	-1.16
13	0.429	2.501	-356322.49	-1.16
15	0.500	2.918	-356322.50	-1.17
17	0.571	3.334	-356322.53	-1.20
18	0.607	3.543	-356322.60	-1.27
19	0.643	3.751	-356322.69	-1.36
21	0.714	4.168	-356323.07	-1.74

Table S2. ζ potentials at different pH and corresponding DLS size.

Sample pH	ζ potential (mV)	DLS size (nm)	DLS-PDI
7.2 (DI water)	36.1 ± 1.2	38.2 ± 12.1	0.15
6 (PBS buffer)	41.6 ± 1.1	55.3 ± 14.6	0.18
5 (PBS buffer)	44.9 ± 1.4	69.0 ± 18.3	0.24
4 (PBS buffer)	52.2 ± 1.2	73.5 ± 16.6	0.29

REFERENCES

(1) Giannozzi, P.; Baroni, S.; Bonini, N.; Calandra, M.; Car, R.; Cavazzoni, C.; Ceresoli, D.; Chiarotti, G. L.; Cococcioni, M.; Dabo, I.; Corso, A. D.; Gironcoli, S. de; Fabris, S.; Fratesi, G.; Gebauer, R.; Gerstmann, U.; Gougoussis, C.; Kokalj, A.; Lazzeri, M.; Martin-Samos, L.; Marzari, N.; Mauri, F.; Mazzarello, R.; Paolini, S.; Pasquarello, A.; Paulatto, L.; Sbraccia, C.; Scandolo, S.; Sclauzero, G.; Seitsonen, A. P.; Smogunov, A.; Umari, P.; Wentzcovitch, R. M. QUANTUM ESPRESSO: A Modular and Open-Source Software Project for Quantum Simulations of Materials. *J. Phys. Condens. Matter* **2009**, *21* (39), 395502. https://doi.org/10.1088/0953-8984/21/39/395502.

(2) Giannozzi, P.; Andreussi, O.; Brumme, T.; Bunau, O.; Nardelli, M. B.; Calandra, M.; Car, R.; Cavazzoni, C.; Ceresoli, D.; Cococcioni, M.; Colonna, N.; Carnimeo, I.; Corso, A. D.; Gironcoli, S. de; Delugas, P.; DiStasio, R. A.; Ferretti, A.; Floris, A.; Fratesi, G.; Fugallo, G.; Gebauer, R.; Gerstmann, U.; Giustino, F.; Gorni, T.; Jia, J.; Kawamura, M.; Ko, H.-Y.; Kokalj, A.; Küçükbenli, E.; Lazzeri, M.; Marsili, M.; Marzari, N.; Mauri, F.; Nguyen, N. L.; Nguyen, H.-V.; Otero-de-la-Roza, A.; Paulatto, L.; Poncé, S.; Rocca, D.; Sabatini, R.; Santra, B.; Schlipf, M.; Seitsonen, A. P.; Smogunov, A.; Timrov, I.; Thonhauser, T.; Umari, P.; Vast, N.; Wu, X.; Baroni, S. Advanced Capabilities for Materials Modelling with Quantum ESPRESSO. *J. Phys. Condens. Matter* **2017**, *29* (46), 465901. https://doi.org/10.1088/1361-648X/aa8f79.

(3) Yan, Y.; Zheng, S.; Xiong, L.; Wang, F.; Cheng, J.; Li, F.; Xiao, Z. First-Principles Calculations of Structural, Electronic and Optical Properties of $ZnGa_2O_4$:Cr^{3+} System. *J. Alloys Compd.* **2022**, *890*, 161862. https://doi.org/10.1016/j.jallcom.2021.161862.

(4) Chen, M.-I.; Singh, A. K.; Chiang, J.-L.; Horng, R.-H.; Wuu, D.-S. Zinc Gallium Oxide—A Review from Synthesis to Applications. *Nanomaterials* **2020**, *10* (11), 2208. https://doi.org/10.3390/nano10112208.
